# Integrated microRNA-mRNA analysis revealing the potential roles of microRNAs in tongue squamous cell cancer

**DOI:** 10.3892/mmr.2015.3467

**Published:** 2015-03-11

**Authors:** XIAO-LI ZHOU, JUN-HUA WU, XIN-JUAN WANG, FU-JUN GUO

**Affiliations:** 1 Department of Stomatology, The First Affiliated Hospital of Henan University of Science and Technology, Luoyang, Henan 471003, P.R. China; 2 Department of Prosthodontics, School of Stomatology, Tongji University, Shanghai 200072, P.R. China

**Keywords:** tongue squamous cell carcinoma, microRNA expression, mRNA expression, Gene Ontology analysis, pathway analysis

## Abstract

Tongue squamous cell carcinoma (TSCC) is a rare and aggressive type of cancer, which is associated with a poor prognosis. Identification of patients at high risk of TSCC tumorigenesis may provide information for the early detection of metastases, and for potential treatment strategies. MicroRNA (miRNA; miR) and mRNA expression profiling of TSCC tissue samples and normal control tissue samples were obtained from three Gene Expression Omnibus (GEO) data series. Bioinformatics analyses, including the Gene Ontology (GO) and Kyoto Encyclopedia of Genes and Genomes were used to identify genes and pathways specifically associated with miRNA-associated TSCC oncology. A total of 25 miRNAs and 769 mRNAs were differentially expressed in the two groups assessed, and all the differentially expressed miRNA and mRNA target interactions were analyzed. The miRNA target genes were predominantly associated with 38 GO terms and 13 pathways. Of the genes differentially expressed between the two groups, and confirmed in another GEO series, miRNA-494, miRNA-96, miRNA-183, runt-related transcription factor 1, programmed cell death protein 4 and membrane-associated guanylate kinase were the most significantly altered, and may be central in the regulation of TSCC. Bioinformatics may be used to analyze large quantities of data in microarrays through rigorous experimental planning, statistical analysis and the collection of complete data on TSCC. In the present study, a novel differential miRNA-mRNA expression network was constructed, and further investigation may provide novel targets for the diagnosis of TSCC.

## Introduction

Oral squamous cell carcinoma (OSCC) is responsible for 24% of all cases of head and neck cancer (1). Despite advances in multimodality treatment, the overall prognosis for patients with OSCC, particularly tongue cancer, has changed little in the last three decades (2). Furthermore, the reasons for variability in the clinical progression of patients with tongue squamous cell cancer (TSCC) remain to be elucidated. Identification of novel prognostic factors may enable the rational selection of appropriate therapeutic options for individual patients. Transcriptional profiling by DNA microarray analysis is an effective tool in cancer research, significantly improving current knowledge regarding tumor development and progression (3). It has also assisted in identifying novel treatment targets and to generate prediction models for prognosis and treatment response (4–6). The cellular and molecular heterogeneity of OSCC, and the numerous genes potentially involved in its development, reiterate the importance of investigating gene alterations on a global scale.

MicroRNAs (miRNAs) are a class of non-coding RNAs, which are between 21 and 25 nucleotides in length and function as regulators of gene expression. Mature miRNAs and Argonaute proteins form the RNA-induced silencing complex, which mediates post-transcriptional gene silencing through induction of mRNA degradation or the inhibition of translation (7). It has previously been estimated that one third of the genes in the human genome are regulated by miRNAs (8), and >1,800 miRNAs have been identified in miRBase version 20.0 (9). miRNAs are involved in several key cellular processes, including apoptosis, proliferation and differentiation (10). Dysregulaton in the expression of miRNA or miRNA mutations result in a gain or loss of miRNA function, which leads to downregulation or upregulation of the target protein. miRNAs have also been found to function as oncogenes or tumor suppressors (11,12).

However, there have been few reports regarding the role of miRNAs in the regulation of TSCC. Furthermore, the regulation of miRNAs and corresponding target mRNAs during the occurrence and development of TSCC remains to be elucidated. The introduction of genome-wide technologies, including gene expression microarrays, has made it possible to achieve a comprehensive view of the alterations of miRNAs and mRNAs involved in TSCC. In addition, the use of bioinformatics enables the analysis of the differentially expressed miRNAs and mRNAs.

The present study aimed to identify the miRNAs and mRNAs involved in the molecular changes associated with TSCC. Published gene expression microarray databases of miRNAs and mRNAs were examined, in order to discriminate between the expression profiles of TSCC samples and normal control samples. The aim was to determine whether the regulation of miRNAs is involved in the pathophysiology of TSCC, and identify novel mechanisms and targets for cancer therapy.

## Patients and methods

### Patients

Microarray data were obtained from three datasets, which consisted of 18, 57 and 38 appropriate samples, respectively. The miRNA microarray series contained data from 15 tumor samples and three healthy control samples, the mRNA microarray test series contained data from 26 tumor samples and 12 healthy control samples, and the mRNA microarray confirmation series contained data from 37 tumor samples and 20 healthy control samples. The three series were accessed at the National Centers for Biotechnology Information (NCBI) Gene Expression Omnibus (GEO) database (http://www.ncbi.nlm.nih.gov/geo/), and the accession numbers were GSE28100, GSE9844 and GSE13601, respectively. Details of the sample features have been presented in previous reports (13–15). The raw values of miRNAs were collected from microarray data and normalized by logarithmic transformation for the ease of further calculation.

### Differentially expressed miRNAs

Differentially expressed miRNAs between the TSCC and normal control samples were identified using the limma method. The P-value and false discovery rate (FDR) were calculated for each differentially expressed miRNA. A threshold was set at fold-change>4, P<0.01 and FDR<0.01, from which the TSCC-associated differentially expressed miRNAs were selected. Unsupervised hierarchical clustering was performed with Cluster software, version 3.0 (Eisen Lab, Stanford, CA, USA) using Pearson’s correlation distance metric and average linkage. The cluster was visualized using Treeview software (Eisen Lab) (16).

### Differentially expressed mRNAs

The differentially expressed mRNAs between the TSCC and normal control samples were identified using the limma method (17). The P-value and the fold-change were calculated for each differentially expressed mRNA. Thresholds were set at P<0.01 and FDR<0.01, from which the TSCC-associated differentially expressed mRNAs were selected. Unsupervised hierarchical clustering was performed with Cluster software using Pearson’s correlation distance metric and average linkage, followed by visualization using Treeview software.

### Gene Ontology (GO) analysis

GO analysis was used to analyze the predominant functions of the differentially expressed genes according to the GO, which is the key functional classification of the NCBI (18,19). Fisher’s exact test and *χ*^2^ test were used to classify the GO category, and the FDR (20) was calculated to correct the P-value; the smaller the FDR, the smaller the error in judging the P-value. The FDR was defined as *FDR* = 1 - *N_k_ / T*, where *N_k_* refers to the number of Fisher’s test P-values less than the *χ*^2^ test P-values. P-values were calculated for the GO terms of all the differentially expressed genes. Enrichment provides a measure of the significance of the function; as the enrichment increases, the corresponding function is considered more specific, enabling identification of the GO terms with more significant functions (21).

### Pathway analysis

Pathway Analysis (The Intelligent Systems and Bioinformatics Laboratory, Detroit, MI, USA) was used to identify the significant pathways associated with the differentially expressed genes, according to Kyoto Encyclopedia of Genes and Genomes (KEGG; http://www.genome.jp/kegg/), Biocarta (http://www.biocarta.com/) and Reatome (http://www.reactome.org/). Fisher’s exact test and *χ*^2^ test were used to select the significant pathways, and the threshold of significance was defined by the P-value (<0.05) and FDR (<0.05) (22–24).

### Annotation of the miRNA targets

The target mRNAs of the differentially expressed miRNAs were predicted based on TargetScan (http://www.targetscan.org/) version 5.2. TargetScan predicts the biological targets of miRNAs by identifying conserved 8mer and 7mer sites, which match the seed region of each miRNA (8). Sites containing mismatches in the seed region, which are compensated by conserved 3′ pairing, are also identified (25). In mammals, predictions are ranked based on the predicted efficacy of targeting, as calculated using the context scores of the site alignments (26,27). TargetScan Human considers matches to annotate human untranslated regions and their orthologs, as defined by UCSC whole-genome alignments. Conserved targeting is also detected within open reading frames.

### miRNA-GO network

The miRNA-GO network was generated according to the association between significant GO terms and miRNAs. The adjacency matrix of miRNA and GO terms: A=(a_i,j_) was calculated from the association between the GO terms and the microRNAs, where a_i,j_ represents the association weight of GO (i) and microRNA (j). In the miRNA-GO network, squares represent microRNAs and circles represent GO terms, and their association is represented by one edge. The center of the network was defined by the degree, which was the contribution that one microRNA made to the surrounding GO terms, or the contribution that one GO term made to the surrounding microRNAs. The key microRNA and GO term in the network always have the highest degrees (28).

### miRNA-mRNA network

The association between differentially expressed miRNAs and mRNAs were calculated by their differential expression values. The network was generated, according to the interactions between the miRNAs and mRNAs listed in the Sanger microRNA database (http://www.mirbase.org/). The adjacency matrix of the miRNA and mRNAs was calculated using A=(a_i,j_), as described above, where a_i,j_ represents the association weight of the mRNA (i) and miRNA (j). The degree was defined as the contribution one miRNA made to the surrounding mRNAs, or the contribution one gene made to the surrounding miRNAs. The key miRNA and gene in the network always have the highest degrees.

### Statistical analysis

The numerical data are presented as the mean ± standard deviation. Differences between the means were analyzed using Student’s t-test. All statistical analyses were performed using SPSS version 13.0 software (SPSS, Inc,. Chicago, IL, USA).

## Results

### Overview of the miRNA expression profiles

**Figure 1:**
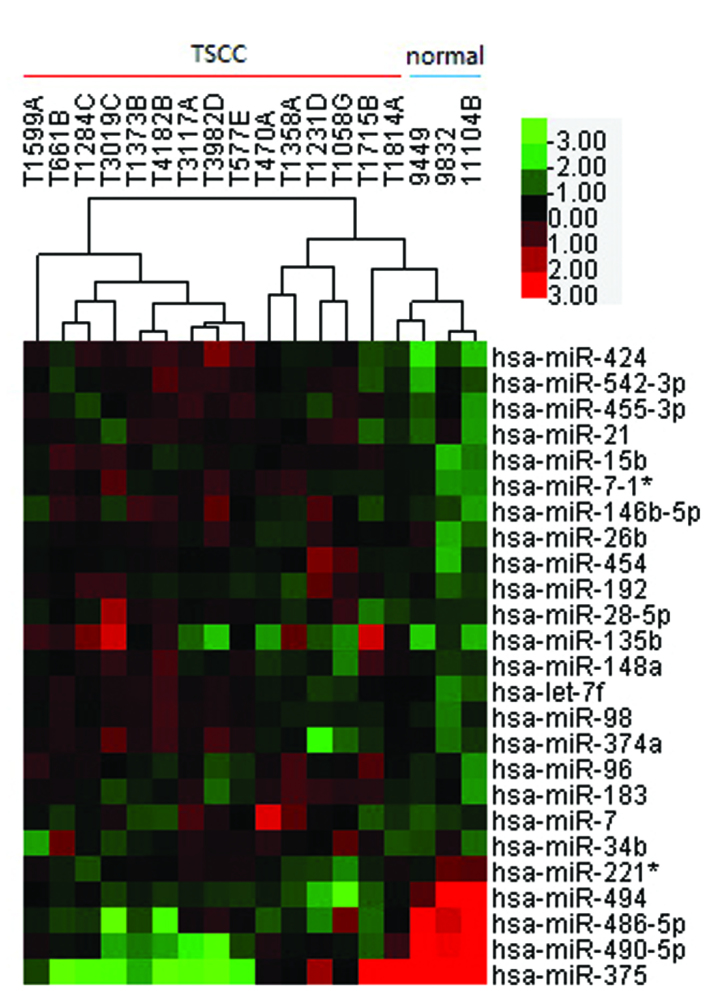
Unsupervised classification of TSCC and normal control samples based on miRNA expression profiling. The miRNA expression data are depicted as a data matrix, in which each row represents a probe and each column represents a sample. The expression levels are depicted according to the color scale (top right). Red and green indicate expression levels above and below the median, respectively. The magnitude of deviation from the median is represented by color saturation. TSCC, tongue squamous cell carcinoma. miR, microRNA.

From the miRNA expression profiles, differentially expressed miRNAs were identified between the TSCC and normal control samples. The miRNA expression profiles in the TSCC samples were determined by calculating the log fold-change TSCC/normal. Since the sample size was limited, the fold-change, FDR and P-values were calculated from the normalized expression values and 26 results were obtained. According to the miRBase (http://www.mirbase.org/) database, miRNA-923 was observed to not be a true miRNA, thus was excluded from further investigation. Therefore, 25 differentially expressed human miRNAs were identified between the 15 TSCC patients and three normal controls. A heat map, constructed using unsupervised hierarchical clustering analyses with threshold values set at fold-change >2, P<0.01 and FDR<0.01, demonstrated that there were 21 overexpressed and four underexpressed miRNAs in the TSCC samples, compared with the normal tissue samples (Fig. 1). According to the FDR values, miR-424, miR-542-3p and miR-454 were the most upregulated and miR-494, miR-490-5p and miR-486-5p were the most downregulated miRNAs, compared with the normal tissue samples (Table I). Upregulated miRNAs were more common, compared with downregulated miRNAs in the TSCC group.

### Overview of the mRNA expression profiles

**Figure 2:**
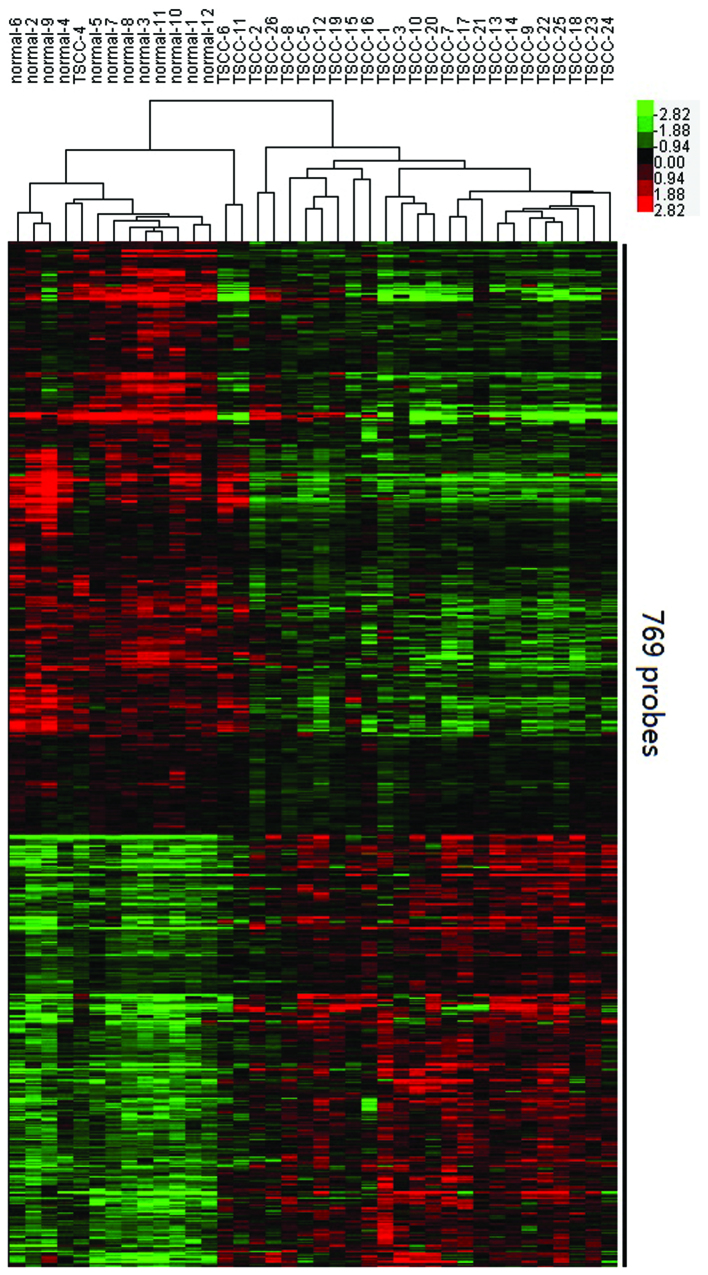
Unsupervised classification of TSCC and healthy control samples based on gene expression profiling. The classification of 38 TSCC and normal control samples from GSE9844 revealed 769 differentially expressed probe sets. Expression data are depicted as a data matrix, in which each row represents a gene and each column represents a sample. Expression cluster 1 contained all 12 normal control samples and three TSCC samples, whereas expression cluster 2 contained 23 of the 26 TSCC samples. The expression levels are depicted according to the color scale (top right). Red and green indicate expression levels above and below the median, respectively. The magnitude of deviation from the median is represented by color saturation. TSCC, tongue squamous cell carcinoma.

In the mRNA microarray test group, ≤54,675 coding transcripts were detected in the 38 samples, using the Affymetrix U133 plus 2.0 array (Affymetrix, Santa Clara, CA, USA). Using the limma method, with a cut-off value of FDR<0.01 between the two groups, 324 probes were upregulated in the TSCC samples and 445 were downregulated. Global mRNA expression patterns were evaluated using hierarchical clustering. The most differentially expressed mRNAs revealed two major clusters, which correlated with the differentiation state of the tumor (Fig. 2). Expression cluster 1 contained all 12 normal control samples and three TSCC samples, whereas expression cluster 2 contained 23 of the 26 TSCC samples. Matrix metalloproteinase 1 was the most significantly upregulated mRNA, and tenascin XB was the most significantly downregulated mRNA (Table II). Downregulated mRNAs were more common than upregulated mRNAs in the TSCC group.

### Microarray-based GO analysis

**Figure 3:**
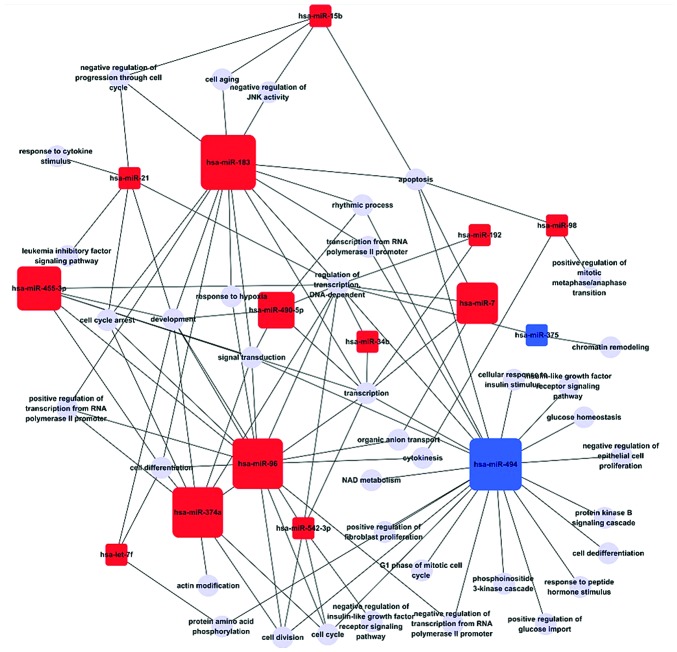
miR-GO interaction network of tongue squamous cell carcinoma. Blue represents downregulation and red represents upregulation. Square nodes represent miRNAs and circular nodes represent GO terms. miR, microRNA; GO, Gene Ontology.

The target mRNAs of the differentially expressed miRNAs were predicted using TargetScan (http://www.targetscan.org/). A total of 5,208 associations between the mRNAs and miRNAs were observed. The intersection set for the predicted target mRNAs and the differentially expressed mRNAs identified from the GSE13601 dataset were selected. Following negative correlation analysis, the eligible mRNAs underwent GO analysis. P<0.01 was considered to indicate GO terms, which were significantly regulated by the differentially expressed miRNAs. The characteristics and associations between the miRNAs and mRNAs are listed in Table III. The highly enriched GO terms targeted by the miRNAs, included regulation of transcription, development and cell differentiation The maximum-enriched GO term was signal transduction, which is a known to be a basic function of miRNA. An miRNA-GO network was constructed to indicate the GO terms, which function in the regulation of TSCC (Fig. 3). In the network, miRNA-494, miRNA-183 and miRNA-96 were found to be central in regulating the majority of the GO terms.

### Microarray-based pathway analysis

**Figure 4:**
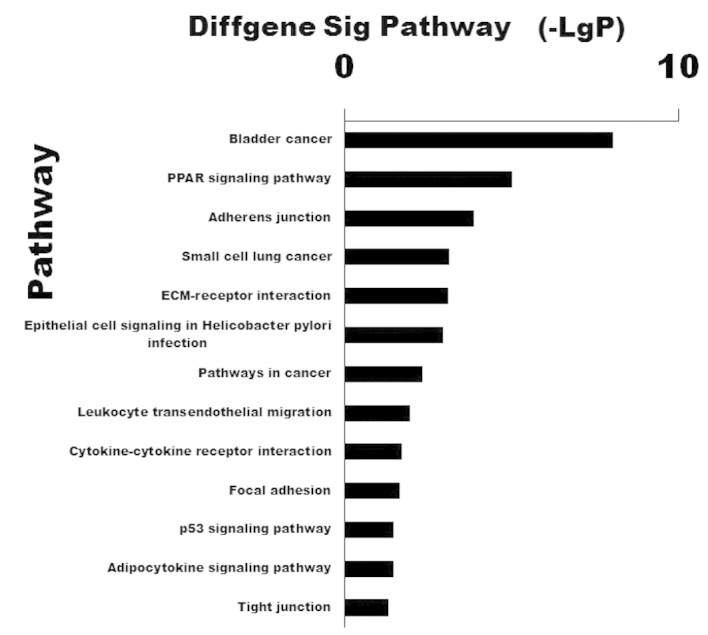
Histogram of signaling pathways that were significantly different between the tongue squamous cell carcinoma and normal control samples. The X-axis indicates the-LgP of the specific pathway. The higher the -LgP, the lower the P-value. -LgP, negative logarithm of the P-value.

The results of the present study suggested that signal transduction and other GO terms may be involved in TSCC, therefore, associated pathways were analyzed, according to the functions and interactions of the differentially expressed genes. Pathway analysis considers relative change direction and fold-change, and the threshold of significance is P<0.05 (29). Using pathway analysis, 14 significant pathways were identified (Fig. 4). The highly enriched pathways targeted by dysregulated mRNAs were: Peroxisome proliferator-activated receptor (PPAR) signaling pathway, adherens junction and extracellular cell membrane (ECM)-receptor interaction. These results suggested that miRNAs may regulate the oncogenesis of TSCC through these pathways.

### MicroRNA-mRNA network

**Figure 5:**
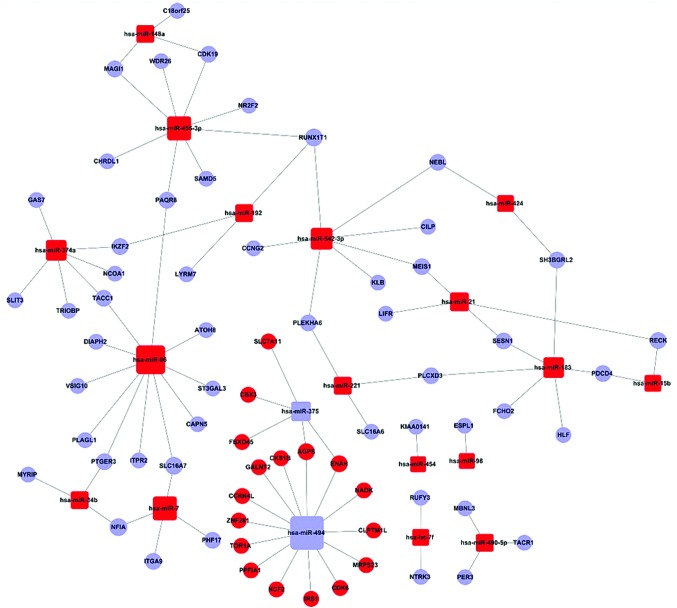
miR-mRNA interaction network of tongue squamous cell carcinoma. Blue represents downregulation and red represents upregulation. Circular nodes represent mRNA and square nodes represent miRNA.

The overlapping mRNAs from the TargetScan predictions and the results of the mRNA microarray of differentially expressed mRNAs in the GO and pathway analyses were selected. The miRNA-mRNA regulatory networks based on these mRNAs were used to identify the putative target mRNAs of the overexpressed and underexpressed miRNAs (Fig. 5). The total number of mRNAs and miRNAs in the network were 68 and 19, respectively. The associations between the miRNAs and the mRNAs are listed in Table IV. In the network, circular nodes indicated mRNAs, square nodes were miRNAs, and edges between two nodes indicated the interactions between the miRNAs and mRNAs. The degree represents the number of target genes regulated by a certain miRNA. The higher the degree, the more central the miRNA occurs within the network. Three dysregulated miRNAs (miR-494, miR-96, and miR-455-3p) had the highest number of target mRNAs. In addition, runt-related transcription factor 1 (RUNX1T1), alkylglycerone phosphate synthase and cyclin-dependent kinase (CDK)19 were targeted by the highest number of miRNAs.

miRNAs may exhibit their central involvement through the above mRNAs, and regulate the formation and development of TSCC. In the present study, another mRNA expression profile data series was used for confirmation of these findings.

**Figure 6:**
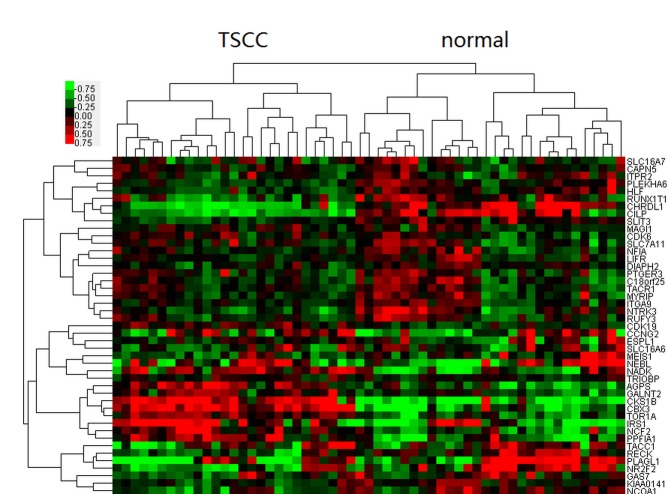
Unsupervised classification of TSCC and healthy control samples based on a cluster of 45 genes. Classification of 57 TSCC and normal control samples from the GSE13601 data series using the 45 differentially expressed mRNAs. Expression data are depicted as a data matrix, in which each row represents a gene and each column represents a sample. The expression levels are depicted according to the color scale (top left). Red and green indicate expression levels above and below the median, respectively. The magnitude of the deviation from the median is represented by color saturation. TSCC, tongue squamous cell carcinoma.

GSE13601 was used as the confirmation data series, which contained 58 samples. The ‘core’ mRNAs in the miRNA-mRNA network were assessed based on the same screening criteria and an associated heat map was constructed. Among the 68 originally identified mRNAs, 45 mRNAs were detected in the GSE13601 data serie using an Affymetrix U95 version 2 array. The TSCC samples were successfully discriminated from the normal control samples in this series (Fig. 6). Furthermore, the fold-changes of these mRNAs in GSE13601 were calculated, and 42 of the 45 mRNAs exhibited the same change direction as the GSE9844 data series. Only SLC7A11, CDK6 and CDK19 exhibited differences.

## Discussion

Despite advances in surgery and radiation therapy, the 5-year survival rate for oral cancer has not improved significantly for several decades and remains at 50–55% (30,31). At present, numerous novel prognostic factors, including cytological features, standard karyotyping, fluorescence *in situ* hybridization, centromeric probes, single nucleotide polymorphism and gene expression profiling are being investigated. Following technical advances and reductions in the cost of gene expression microarrays, they are considered a useful tool for investigating the development and progression of tumors. Owing to the high throughout of microarrays, novel genes that affect the development of TSCC can be identified. miRNAs are regulatory factors, which are considered to be involved in the progression of TSCC and provide a possible target for TSCC therapy (13).

Understanding the clinical relevance of miRNA expression patterns in TSCC is necessary to classify heterogeneous tumors and circumvent the therapeutic challenges faced in their clinical management. However, miRNAs, which indirectly regulate the pathophysiological process of TSCC, and the possible target mRNAs, require elucidation. Microarrays are a useful tool for investigating the development and progression of tumors, owing to their high throughout; however, it remains difficult to predict TSCC, predominantly due to the challenges in interpreting the complex data produced (32) and determining the responsible genes. The present study used bioinformatics to analyze the functions and pathways associated with differentially expressed miRNAs and mRNAs, to further clarify their biological significance to reveal the key miRNAs and possible target mRNAs affecting the formation of TSCC.

The present study identified 26 differentially expressed miRNAs in TSCC compared with normal tongue tissue samples. Since the expression of miRNA is known to be tissue- and tumor-specific (33), using the appropriate subset of tumor samples and the corresponding normal control samples is important to reduce the potential complexities associated with analyzing heterogeneous tumors. The present study aimed to investigate miRNA-mRNA regulation in TSCC, therefore, two gene expression microarray datasets were used to identify the mRNA targets of miRNAs. The GSE9844 dataset was used as the test expression profile, in which 769 differently expressed mRNAs were identified. The mRNAs, which were negatively correlated with the previously identified differentially expressed miRNAs were then used to further investigate the role of miRNAs in TSCC.

The GO is widely recognized as the leading tool for the organization and functional annotation of molecular attributes (34). By using a cut-off value of P<0.01, significant GO terms and associated genes were identified. Guo *et al* (35) previously performed a GO analysis to analyze an miRNA microarray, and revealed that mir-15b and miR-16 may be indispensable for apoptosis through targeting B-cell lymphoma 2. In the present study, GO terms for the transcriptional regulatory response were found to be important in TSCC through the function of miRNAs. This finding was concordant with the predominant biological function of miRNAs in humans. Transcriptional regulation is a major function of miRNAs (36), and the significant changes in this term in the present study further certified the results. Jiang *et al* (37) previously reported that miRNA-7 contributes to the suppression of tumorigenesis in TSCC by targeting insulin-like growth factor 1 through cell cycle arrest (37). In addition, Yao *et al* (38) demonstrated that sulforaphane inhibits hypoxia-inducible factor-1α by activating the c-Jun N-terminal kinase pathway in TSCC (38). Therefore, it was hypothesized that the other miRNAs listed may have functions in the progression of TSCC, which remain to be elucidated.

Pathway analyses can reveal the distinct biological processes and significant pathways associated with the differentially expressed mRNAs. This permits a comprehensive understanding of the interactions of genes, the functions that they are involved in, and associations between upstream and downstream genes. Pathway analyses can also identify genes associated with these significant pathways, which may be regulated by miRNAs. The present study identified pathways regarding PPAR signaling, adherens junctions and p53 signaling, thus confirming their concordance with GO terms and their importance in TSCC. The PPAR signaling pathway has previously been considered a useful prognostic marker and a potential therapeutic target for TSCC (39), however, there have been no reports regarding its molecular mechanism and miRNA regulation. Numerous studies have demonstrated that cytokine interaction is involved in the process of tumor growth, which is important for TSCC (40,41). The miR-34a-sirtuin 6 axis was previously found to be involved in various types of squamous cell cancer, which also demonstrates the impact of the p53 signaling pathway on TSCC (42). There remains a lack of information regarding miRNAs in TSCC or the associated signaling pathway information regulated by miRNAs. The results of the present study suggested that other, apparently irrelevant pathways were be controlled by miRNAs and have functions in TSCC, which requires further investigation. In the present study, the pathway analyses identified equally important roles and functions as the GO analysis.

The present study also investigated the genes associated with significant GO terms and pathways, and 68 genes in common were identified, possibly regulated by miRNAs in TSCC. It has been demonstrated that miRNA-494 is upregulated in whole blood samples of patients with OSCC and may be used as a potential biomarker (43) however, there remains a lack of information regarding its role in TSCC. miRNA-96 and -183 belong to the miRNA-183 family and, in a meta-analysis have been identified as useful prognostic markers and therapeutic targets in various types of cancer (44). The RUNX family includes sequence-specific transcription factors, which are closely associated with various cellular processes, including development, differentiation and/or tumorigenesis, and have been implicated in cancer cells through the miRNA-23a cluster (45). PDCD4 has been also been suggested as a potential marker of TSCC, as selected by a cDNA microarray (46). Membrane-associated guanylate kinase (MAGI1) is a partner of the PDZ-domain and has been also been identified as a serum biomarker of OSCC (47). Although their functions remain to be fully elucidated, several miRNAs may be associated with the regulation of TSCC. In the present study, the second mRNA expression series, used for confirmation, verified the accuracy of the initial results. Based on these data, further investigation of miRNA expression and target functions, and investigation of the regulation of the identified miRNAs and pathway functions are required. This may assist in improving the clinical diagnosis and treatment of patients with TSCC.

In conclusion, the present study identified, by correlating the mRNA and miRNA expression data from two different platforms, putative miRNA-mRNA interactions in TSCC. The miRNA-GO network and the miRNA-pathway analyses identified pathways controlling the PPAR and p53 signaling pathways and adherens junctions, and the focal adhesion and ECM-receptor interaction pathways. Network analysis identified important miRNAs and mRNAs, including miRNA-494, miRNA-96, miRNA-183, RUNX1T1, PDCD4 and MAGI1, which may be involved in the progression of TSCC. The successful verification of these mRNAs in the GSE13601 series provided further evidence that differentially expressed miRNAs in TSCC may regulate tumor formation through regulation of target mRNAs, and may be used to discriminate tumor samples from normal samples. Based on the integrated analysis of transcription features, these results may provide an important contribution to future investigations aimed at characterizing the role of specific miRNAs in the pathogenesis of TSCC, and may contribute to improving diagnosis and treatment.

## Figures and Tables

**Table I:** Differentially expressed miRNAs detected by microarray analysis of TSCC samples.

miRNA	P-value	Fold-change
Downgulated in TSCC		
hsa-miR-490-5p	4.84E-04	0.22
hsa-miR-494	1.54E-04	0.17
hsa-miR-486-5p	2.01E-03	0.21
hsa-miR-375	3.17E-03	0.07
Upregulated in TSCC		
hsa-miR-424	2.36E-06	9.55
hsa-miR-454	7.29E-06	5.50
hsa-miR-542-3p	1.10E-05	5.67
hsa-miR-15b	1.40E-05	6.58

miRNA, microRNA; TSCC, tongue squamous cell carcinoma.

**Table II:** Differentially expressed mRNAs detected by microarray analysis of TSCC samples.

mRNA	P-value	Fold-change
Downregulated in TSCC		
TNXB	1.14E-11	0.20
PADI1	6.44E-11	0.18
SPNS2	2.22E-10	0.27
CD1C	6.93E-10	0.27
GSTM5	1.99E-09	0.34
ADH1B	2.55E-09	0.07
HLF	3.12E-09	0.37
HPGD	4.38E-09	0.11
GCOM1	6.39E-09	0.17
BBIP1	7.96E-09	0.48
Upregulated in TSCC		
MMP1	4.44E-16	123.60
MYO1B	3.10E-11	4.40
IL8	1.64E-10	10.08
PTHLH	5.93E-10	17.09
MYO1B	9.47E-10	5.15
MMP3	1.02E-09	10.48
CDH3	6.25E-09	5.33
KRT17	8.74E-09	13.53
COL4A6	1.08E-08	6.89
CXCL1	1.44E-08	10.85

TSCC, tongue squamous cell carcinoma.

**Table III:** miRNA-gene ontology network characteristics.

miRNA		Gene Ontology	
Name	Degree	Term	Degree
hsa-miR-494	22	Regulation of transcription, DNA-dependent	12
hsa-miR-183	14	Transcription	9
hsa-miR-96	11	Development	8
hsa-miR-374a	10	Signal transduction	6
hsa-miR-21	6	Apoptosis	5
hsa-miR-455-3p	6	Cell differentiation	5
hsa-miR-542-3p	6	Cell cycle	4
hsa-miR-490-5p	5	Cell cycle arrest	4
hsa-miR-15b	4	Cell division	4
hsa-miR-7	4	Negative regulation of progression through cell cycle	3
hsa-let-7f	3	Positive regulation of transcription from RNA polymerase II promoter	3
hsa-miR-98	3	Rhythmic process	3
hsa-miR-192	2	Cell aging	2
hsa-miR-34b	2	Cytokinesis	2
hsa-miR-375	2	Negative regulation of JNK activity	2
		Negative regulation of transcription from RNA polymerase II promoter	2
		Organic anion transport	2
		Protein amino acid phosphorylation	2
		Response to hypoxia	2
		Transcription from RNA polymerase II promoter	2
		Actin modification	1
		Cell dedifferentiation	1
		Cellular response to insulin stimulus	1
		Chromatin remodeling	1
		G1 phase of mitotic cell cycle	1
		Glucose homeostasis	1
		Insulin-like growth factor receptor signaling pathway	1
		Leukemia inhibitory factor signaling pathway	1
		NAD metabolism	1
		Negative regulation of epithelial cell proliferation	1
		Negative regulation of insulin-like growth factor receptor signaling pathway	1
		Phosphoinositide 3-kinase cascade	1
		Positive regulation of fibroblast proliferation	1
		Positive regulation of glucose import	1
		Positive regulation of mitotic metaphase/anaphase transition	1
		Protein kinase B signaling cascade	1
		Response to cytokine stimulus	1
		Response to peptide hormone stimulus	1

miR, microRNA. The degree indicates the contribution of the miRNA to the surrounding GO terms, or of the GO term to the surrounding miRNAs.

**Table IV:** miR-mRNA network characteristics.

Target	Degree
miRNA	
hsa-miR-494	14
hsa-miR-96	11
hsa-miR-455-3p	8
hsa-miR-542-3p	7
hsa-miR-183	6
hsa-miR-374a	6
hsa-miR-375	5
hsa-miR-21	4
hsa-miR-7	4
hsa-miR-148a	3
hsa-miR-192	3
hsa-miR-221	3
hsa-miR-34b	3
hsa-miR-490-5p	3
hsa-let-7f	2
hsa-miR-15b	2
hsa-miR-424	2
hsa-miR-454	1
hsa-miR-98	1
mRNA	
RUNX1T1	3
AGPS	2
CDK19	2
ENAH	2
IKZF2	2
MAGI1	2
MEIS1	2
NEBL	2
NFIA	2
PAQR8	2
PDCD4	2
PLCXD3	2
PLEKHA6	2
PTGER3	2
RECK	2
SESN1	2
SH3BGRL2	2
SLC16A7	2
TACC1	2
ATOH8	1
C18orf25	1
CAPN5	1
CBX3	1
CCNG2	1
CCRN4L	1
CDK6	1
CHRDL1	1
CILP	1
CKS1B	1
CLPTM1L	1
DIAPH2	1
ESPL1	1
FBXO45	1
FCHO2	1
GALNT2	1
GAS7	1
HLF	1
IRS1	1
ITGA9	1
ITPR2	1
KIAA0141	1
KLB	1
LIFR	1
LYRM7	1
MBNL3	1
MRPS23	1
MYRIP	1
NADK	1
NCF2	1
NCOA1	1
NR2F2	1
NTRK3	1
PER3	1
PHF17	1
PLAGL1	1
PPFIA1	1
RUFY3	1
SAMD5	1
SLC16A6	1
SLC7A11	1
SLIT3	1
ST3GAL3	1
TACR1	1
TOR1A	1
TRIOBP	1
VSIG10	1
WDR26	1
ZNF281	1

miR, microRNA; Degree indicates the contribution of the miRNA to the surrounding mRNAs, or of the mRNA to the surrounding miRNAs.
